# The Frequency of Intermediate Alleles in Patients with Cerebellar Phenotypes

**DOI:** 10.1007/s12311-023-01620-7

**Published:** 2023-10-31

**Authors:** Elena Capacci, Silvia Bagnoli, Giulia Giacomucci, Costanza Maria Rapillo, Alessandra Govoni, Valentina Bessi, Cristina Polito, Irene Giotti, Alice Brogi, Elisabetta Pelo, Sandro Sorbi, Benedetta Nacmias, Camilla Ferrari

**Affiliations:** 1 Department of Neuroscience, Psychology, Drug Research and Child Health (NEUROFARBA), University of Florence, Florence, Italy; 2 Neuromuscular-Skeletal and Sensory Organs Department, AOU Careggi, Florence, Italy; 3 IRCCS Fondazione Don Carlo Gnocchi, Florence, Italy; 4 SODc Diagnostica Genetica, Azienda Ospedaliero Universitaria Careggi, Florence, Italy

**Keywords:** Cerebellar ataxia, Intermediate allele, Gray zone, FXTAS, SCA8, SCA2

## Abstract

Cerebellar syndromes are clinically and etiologically heterogeneous and can be classified as hereditary, neurodegenerative non-hereditary, or acquired. Few data are available on the frequency of each form in the clinical setting. Growing interest is emerging regarding the genetic forms caused by triplet repeat expansions. Alleles with repeat expansion lower than the pathological threshold, termed intermediate alleles (IAs), have been found to be associated with disease manifestation. In order to assess the relevance of IAs as a cause of cerebellar syndromes, we enrolled 66 unrelated Italian ataxic patients and described the distribution of the different etiology of their syndromes and the frequency of IAs. Each patient underwent complete clinical, hematological, and neurophysiological assessments, neuroimaging evaluations, and genetic tests for autosomal dominant cerebellar ataxia (SCA) and fragile X-associated tremor/ataxia syndrome (FXTAS). We identified the following diagnostic categories: 28% sporadic adult-onset ataxia, 18% cerebellar variant of multiple system atrophy, 9% acquired forms, 9% genetic forms with full-range expansion, and 12% cases with intermediate-range expansion. The IAs were six in the FMR1 gene, two in the gene responsible for SCA8, and one in the ATXN2 gene. The clinical phenotype of patients carrying the IAs resembles, in most of the cases, the one associated with full-range expansion. Our study provides an exhaustive description of the causes of cerebellar ataxia, estimating for the first time the frequency of IAs in SCAs- and FXTAS-associated genes. The high percentage of cases with IAs supports further screening among patients with cerebellar syndromes.

**Supplementary Information:**

The online version contains supplementary material available at 10.1007/s12311-023-01620-7.

## Introduction

Cerebellar ataxias (CA) are a group of clinically heterogeneous disorders; the causes of which can be categorized as genetic, acquired, or neurodegenerative. Acquired forms include immune-mediated ataxias (i.e., gluten ataxia, paraneoplastic), toxic ataxia (i.e., alcohol-related ataxia), and cerebellar ataxia associated with vitamin deficits (vitamin B12, vitamin E), while non-hereditary degenerative forms are the cerebellar variant of multiple system atrophy (MSA-C) and sporadic adult-onset ataxia (SAOA) [1, 2]. Inherited ataxias can be autosomal recessive, autosomal dominant (SCAs), or X-linked and most of them are caused by trinucleotide repeat expansions [2, 3]. The length of the repeated sequence has a specific pathological threshold for each disease, and when it is above the threshold, the expansion is associated with the disease manifestation. A range of repeats, known as intermediate alleles (IAs) or gray zones, exists that is shorter in number than the pathological range but greater than that most frequently found in the healthy population [4, 5]. IAs are presumed to not cause the disease but they are at high risk of expansion during meiosis and subsequently the offspring of carriers can inherit a pathological allele [4]. In the last few years, researchers reported the association between neurological symptoms and the IAs in the genes responsible for SCAs [6–11] and the IAs in the FMR1 gene responsible for fragile X-associated tremor/ataxia syndrome (FXTAS) [12–18]. The clinical implication of these associations remains uncertain [19, 20]: the clinical phenotypes of patients bearing the IAs often differ from that of patients with the pathological allele; few and controversial data are available on the frequency of IAs among ataxic patients [13–16, 20]. Therefore, the contribution of IAs as a cause of cerebellar syndromes remains unknown, making the diagnostic process of CA more challenging.

In the present study, we analyze a cohort of Italian ataxic patients in order to illustrate the distribution of the different etiologies of CA, estimate the frequency of IAs in SCAs genes and FXTAS gene, describe the phenotypes associated with IAs, and assess the relevance of IAs as a cause of CA.

## Materials and Methods

### Population

The study sample included 66 patients, consecutively recruited among subjects referred for cerebellar signs and symptoms to the Neurology Department of Careggi Hospital, Florence, Italy, from September 2017 to September 2022. Patients in which cerebellar signs and symptoms were found to be due to cerebral ischemia, hemorrhage, neoplasm, or demyelinating disease were excluded. Demographic data included age, sex, age at symptom onset, family history for neurological disorders, and past medical history and treatment. All patients underwent neurological examination including the scale for the assessment and rating of ataxia (SARA) [21] at the first visit and at each control visit. Control visits were performed every 6 months. Hematological screening included thyroid and liver function, vitamin dosage, alpha-fetoprotein, creatine kinase, caeruloplasmin/copper, bile acids, coeliac serology, and complete panel of autoantibodies [22, 23]. All patients underwent brain magnetic resonance imaging (MRI), sensory and motor evoked potentials (SEP and MEP), electromyography (EMG), vestibular and ophthalmological examination, and evaluation of autonomic functions (cardiovascular, urogenital, and gastrointestinal systems). In specific cases, patients also performed total body computed tomography (CT) scan, [123I]FP-CIT single-photon emission computed tomography ([123I]FP-CIT SPECT), or lumbar puncture including measurement of biomarkers of neurodegeneration. Cognitive performance was explored by mini mental state examination (MMSE) [24]. Genetic tests performed on the 66 patients included genes responsible for SCA1, 2, 3, 6, 7, 8,1 2, 17, DRPLA, and FXTAS. In specific cases, FXN (frataxin), SPG7, and frontotemporal dementia-related genes were also tested. Genetic screening (SCA1, 2, 3, 6, 7, 8, 12, 17, DRPLA, FXTAS) was also performed in a control cohort of 70 healthy subjects, 33 males and 37 females, with a mean age of 77.6 (± 4.5) years. All healthy participants underwent a clinical evaluation in order to exclude the presence of any neurological disorder.

### Categories

We divided the patients into the following diagnostic categories: acquired ataxia, MSA-C, CANVAS, full-range expansion, expansion in the gray zone, SAOA, and unknown etiology. Acquired forms included toxic causes, vitamin deficits, and immune-mediated cerebellar ataxia. The category of MSA-C included only patients that fit diagnostic criteria for probable MSA [25]. Patients with CANVAS fit the criteria for clinically defined and clinically probable forms [26]. Full-range expansion and gray zone expansion are classified based on the lengths of repeats reported in the literature and are illustrated in Table 1 [4, 5, 27–31]. Once all previous diagnoses had been ruled out, patients were classified as affected by SAOA in case of negative family history for ataxia, progressive cerebellar impairment present for at least 3 years, and cerebellar atrophy detected by brain MRI [2, 32]. The remaining patients were labeled as affected by ataxia of unknown etiology. In this last group, subjects with a probable genetic disorder in which the causative gene had not been identified were included as well as patients with sporadic ataxia that did not fit the other diagnostic criteria.

**Table 1:** Normal, intermediate, and pathological numbers of triplets for each genetics form [4, 5, 27–31]

Disease	Normal	Intermediate	Pathological
SCA1 (CAG)n	6–35	36–38	39–91
SCA2 (CAG)n	14–31	32–33	34–500
SCA3 (CAG)n	11–44	45–59	60–87
SCA6 (CAG)n	4–18	19	20–33
SCA7 (CAG)n	4–27	28–33	34–460
SCA8 (CTG)n	15–50	50–80	80–250
SCA12 (CAG)n	6–32	40–49	51–78
SCA17 (CAG)n	25–40	41–44*	41–66
DRPLA (CAG)n	3–38	39–47	48–93
FMR1 (CGG)n	5–44	45–54	55–200 (premutation)

### Genetic Analysis

Total DNA was isolated from peripheral blood using standard methods (QIAamp DNA Blood Mini QIAcube kit). DNA quality was tested with a NanoDrop ND-3300® Fluorospectrometer. DNA samples were stored at + 4 °C until use. A polymerase chain reaction (PCR) amplification assay was performed to determine trinucleotide repeat expansion SCA genes. The forwarded primer was modified with 6-carboxyfluorescein (6-FAM), a fluorescent dye for labeling oligonucleotides [33]. The size of the fragment was determined by capillary electrophoresis using a SeqStudio automated DNA sequencer and the GeneMapper version 4.0 software (Applied Biosystems). AmplideX® mPCR *FMR1* Kit (Asuragen) was used to determine the FXTAS CGG repeat expansions.

### Statistical Analysis

The demographic-clinical features of patients were described by mean and standard deviation in case of continuous variables, and by percentage in case of categorical variables. Due to the identification of genetic expansions (full and intermediate range) in the study population, we included a control cohort to compare the results of the genetic analysis. We performed the Shapiro–Wilk test to explore the distribution of the genetic variables in the two cohorts. The variables were not normally distributed; thus, the Fisher exact test was used to compare the frequency of genetic expansions of the two cohorts.

## Results

**Fig. 1:**
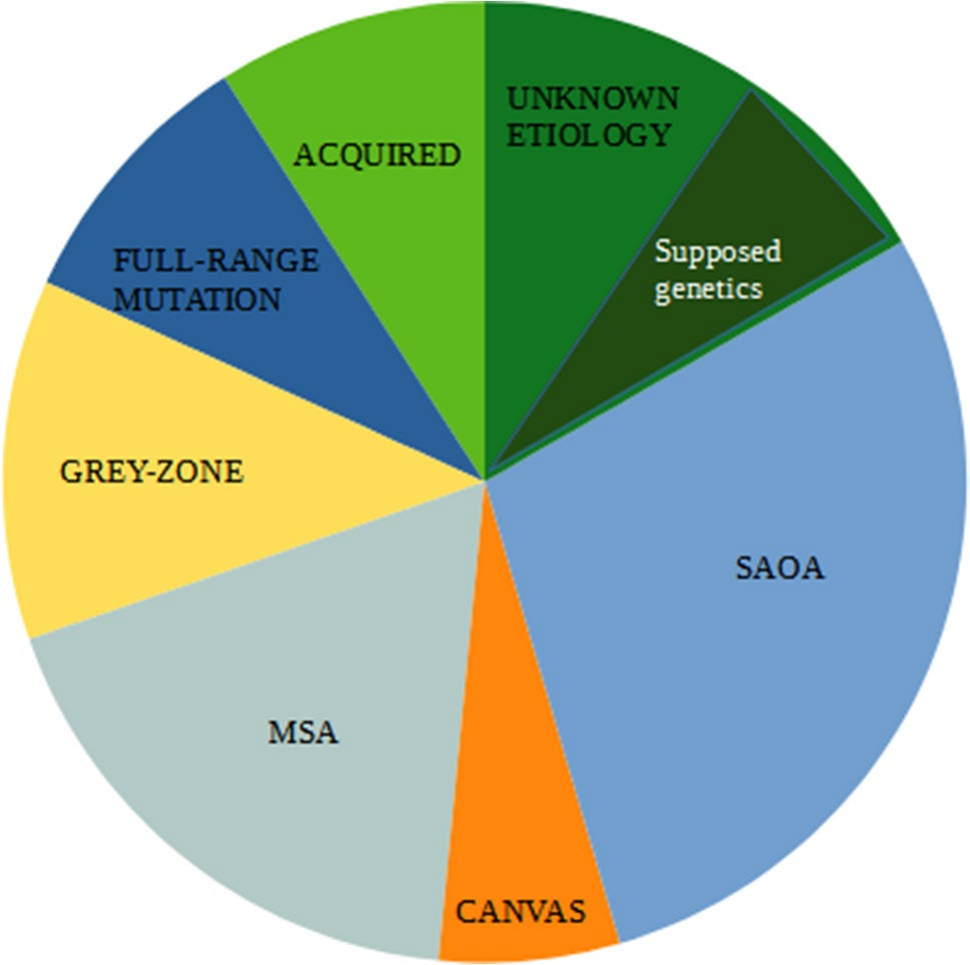
Distribution of the diagnostic categories in the study population (*n* = 66)

The study sample included 66 patients, 29 males and 37 females with a mean age of 57.12 years. The mean follow-up time was 2.6 years, with a minimum of 6 months. Based on clinical, instrumental, and genetic tests, the following diagnoses were made (Fig. 1): 12 MSA-C (18.18%); six acquired ataxias (9.09%) including three immune-mediated forms, of which is one gluten ataxia with celiac disease, one post-infectious cerebellitis associated with SARS-CoV-2, one paraneoplastic ataxia associated with ovarian cancer, two alcohol-related cerebellar degeneration, and one hypomagnesemia-induced cerebellar ataxia in alcohol assumption; six genetic forms with full-range expansion (9.09%) in particular two males with SCA2, one male with FXTAS, two patients with Friedreich ataxia, and one patient with SPG7; eight patients bearing IAs (12.12%), in particular six carrying the IA in FMR1 gene and two the IA in the SCA8 gene; one of the patients with FXTAS gray zone allele was also a carrier of IA in ATXN2-gene; four CANVAS (6.06%); 19 SAOA (28.79%); and 11 cases were classified as unknown etiology (16.67%) of which are five probable genetic forms with unidentified gene and six patients that did not meet any other criteria. The demographic characteristics of the study population are illustrated in Table 2. In the control cohort, we identified one IA on the FMR1 gene, while the length of the other screened genes resulted in the normal range in all subjects. The carrier of the IA-FMR1 was an 81-year-old female, bearing an allele with 48 triplets and one with 29. The frequency of IA on the FMR1 gene in the study population (9.09%) was higher than that found in the control cohort (1.42%), *p* 0.047.

**Table 2:** Demographic and genetics characteristics of the study population (*n* = 66) by diagnosis

Features	MSA-C (12)	Acquired (6)	Full-range-expansion (6)	Intermediate alleles (8)	SAOA (19)	CANVAS (4)	Unknown etiology (11)
Age at onset, years (SD)	65.2 (6.9)	60 (13.3)	36.8	55.75 (12.5)	59.9 (14)	66 (7.8)	46 (15.5)
Age, years (SD)	67.41 (16.5)	61.6 (10.2)	48.3 (16.1)	59.2 (16.8)	63.50 (12.7)		50.63 (10.1)
Female, sex (%)	5 (41.6%)	5 (83.3%)	2 (33.3%)	6 (75%)	11 (57.9%)	0	8 (72.7%)
Family history (%)	1 (8.3%)	1 (16.6%)	4 (66.6%)	2 (25%)	0	0	5 (45.5%)
SCA1 mean (SD)	28.25 (0.7)	28.5 (0.7)	28 (0.6)	28.67 (1.7)	29.17 (2.1)	29.75 (0.9)	28.75 (1.7)
SCA2 mean	20.13 (0.9)	19.5 (0.7)	23.3 (0.4)	22	20.78 (1.1)	21.25 (0.5)	20 (1.1)
SCA3 mean (SD)	19.13 (2.2)	16.5 (0.7)	21.2 (1.7)	19.17 (0.8)	16.72 (4.1)	19.75 (2.5)	17.56 (2.6)
SCA6 mean (SD)	10.11 (1.4)	9 (1.4)	11.4 (2)	10.67 (1.5)	11.22 (2.3)	11.75 (0.5)	9.33 (3.1)
SCA7 mean (SD)	4.83 (2.2)	4.5 (0.7)	5.1 (2.1)	6.4 (1.1)	5.71 (1.9)	6 (0.8)	5.57 (0.9)
SCA8 mean (SD)	22.88 (1.1)	25.5 (2.1)	24.6 (1.8)	29	24.4 (4)	24 (0.1)	25.33 (2.1)
SCA12 mean (SD)	13.43 (0.9)	11 (1.4)	10.9 (1.0)	12.5 (1.8)	12.29 (4.1)	12.5 (2.3)	12.13 (2.7)
SCA17 mean (SD)	36.33 (1)	36 (0.6)	37.2 (0.9)	35.83 (0.7)	36.12 (1.1)	37.25 (1.8)	35.89 (1.3)
DRPLA mean (SD)	13.29 (0.9)	12.5 (0.7)	12.45 (0.9)	13.67 (1.03)	14.41 (3.7)	12 (4.5)	13.13 (2.6)
FMR1 mean (SD)	29.63 (3.9)	30.5 (0.7)			29.41 (2.8)	30.25 (0.5)	33.75 (3.5)

### Patients with Intermediate Alleles (for Detailed Description of Cases, See Supplementary Materials)

#### FXTAS (45–54 CGG Repeats) [29]

**Fig. 2:**
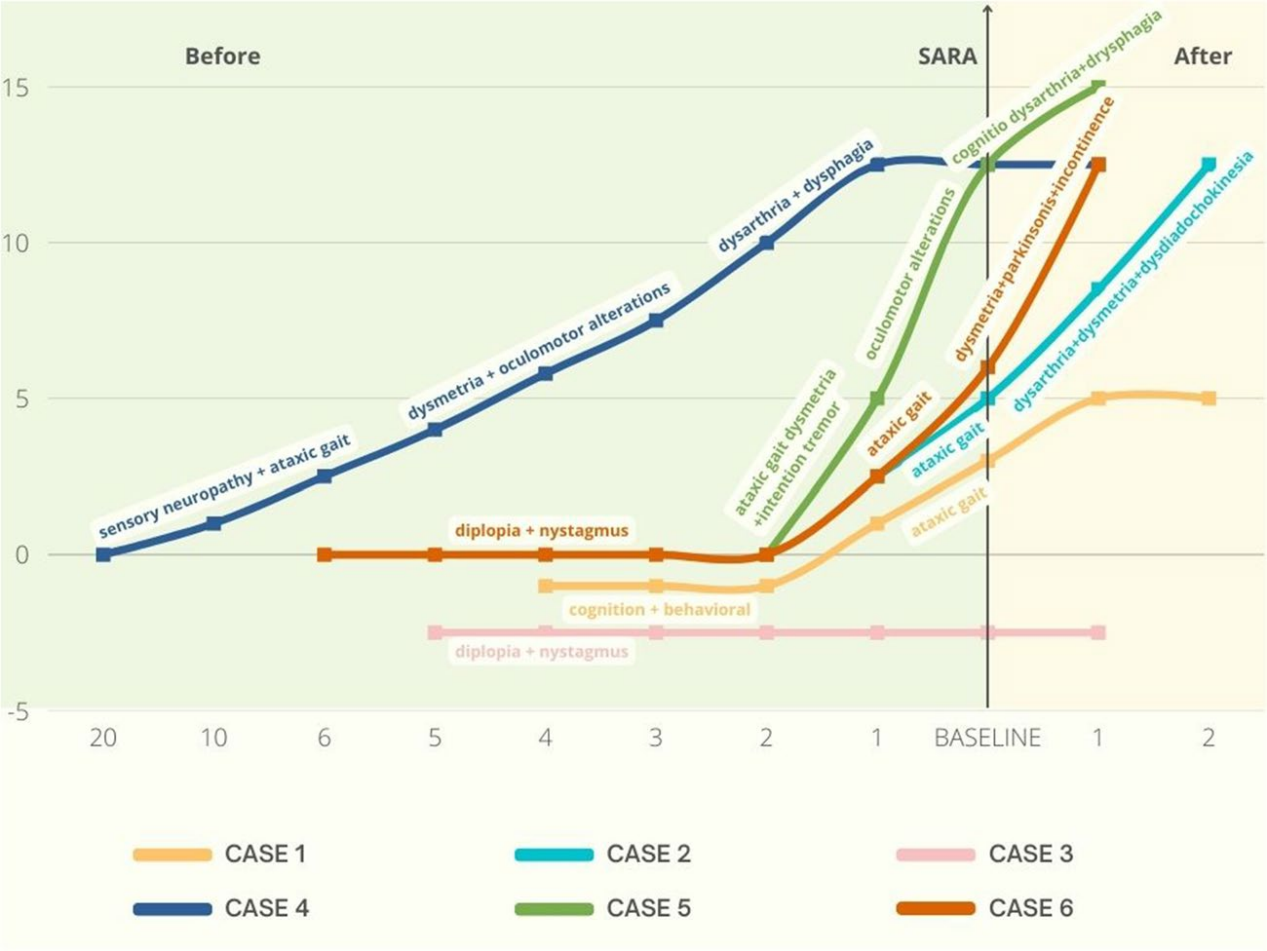
Clinical presentation and disease course in the six FMR1-intermediate allele carriers. Case 1 (yellow line) (45 triplets + ATXN2-IA): total disease duration is 5 years. The disease started 4 years before baseline visit with cognitive and behavioral disorder. Ataxic gait started 1 year before baseline visit. At baseline visit, she scored 4 at SARA. Progression of cerebellar symptoms was mild and the patient got 5 at SARA after 1-year follow-up. Case 2 (light blue line) (48 triplets): total disease duration is 4 years. The disease started 2 years before baseline visit with ataxic gait. SARA score was 5 at baseline visit. During the 2 years of follow-up, the patient also presented dysarthria, dysmetria, and dysdiadochokinesia. The disease worsened rapidly, and the SARA score was 12 at the last follow-up. Case 3 (pink line) (45 triplets): total disease duration is 6 years. The patient presented only diplopia and nystagmus. SARA score was 0 till the last follow-up. Case 4 (blue line) (46 triplets): total disease duration is 21 years. The disease started 20 years before baseline visit with ataxic gait and sensory neuropathy, followed by dysmetria, oculomotor alterations, dysarthria, and dysphagia. SARA score at baseline was 12, and stable in the follow-up period. Case 5 (green line) (52 triplets): total disease duration is 3 years. The disease started 2 years before baseline visit with ataxic gait followed by dysmetria, intention tremor, oculomotor alteration, dysarthria, dysphagia, and cognitive decline. The disease progressed rapidly, and after 2 years from symptoms onset, SARA score was 13. At the last follow-up, 6 months later, SARA score was 14. Case 6 (orange line) (54 triplets): total disease duration is 8 years. The disease started 6 years before baseline visit with diplopia and nystagmus. Four years later, the patient presented also ataxic gait, followed by dysmetria, parkinsonism, and urinary incontinence. SARA score at baseline was 17, and 18 at the last follow-up

**Fig. 3:**
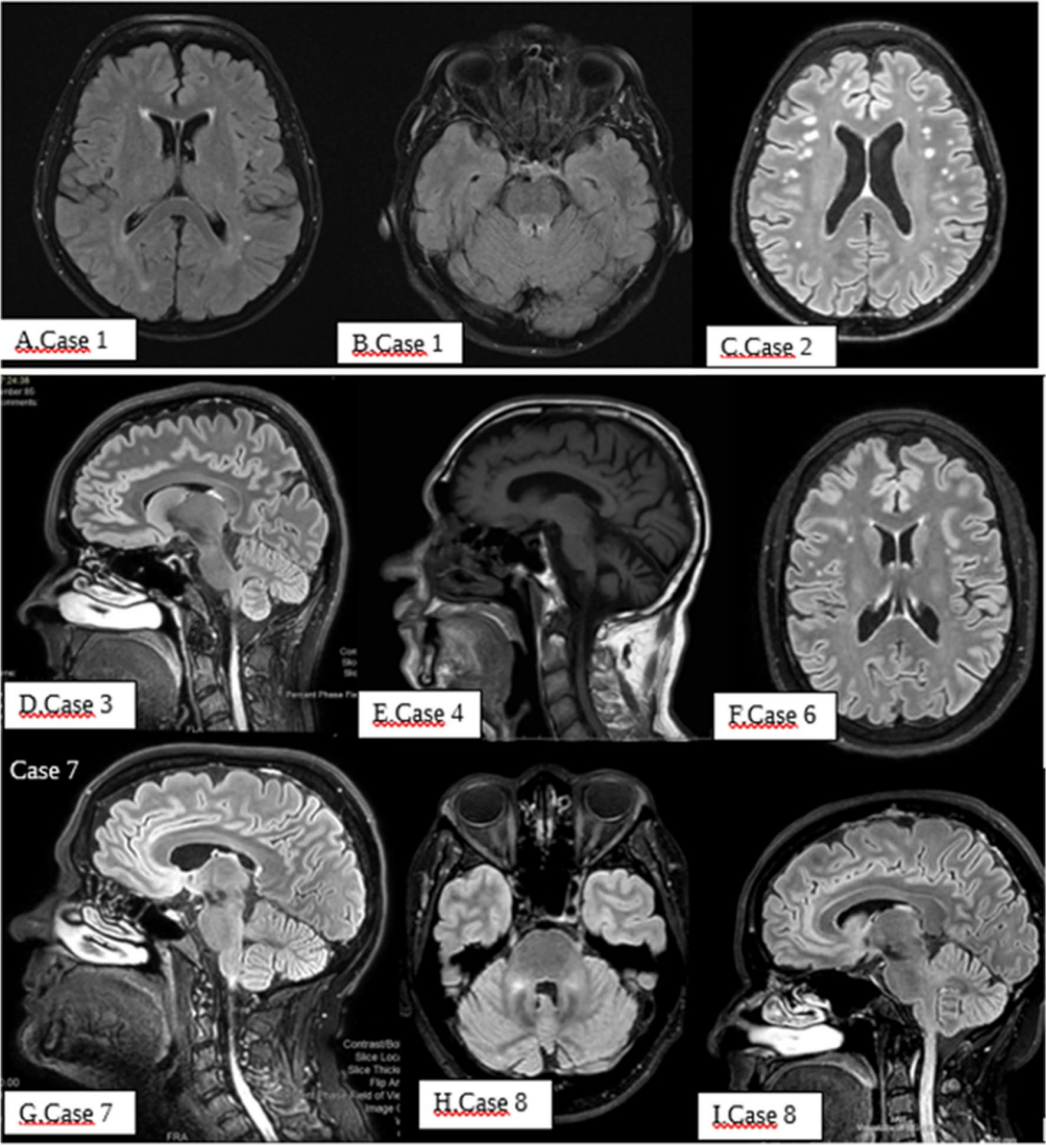
Neuroimaging abnormalities in patients carrying the intermediate alleles. Case 1 (**A**–**B**): axial FLAIR-weighted MRI showing hyperintensity of the subcortical white matter (**A**) and of the superior cerebellar peduncles with extension to the posterior portion of the midbrain (**B**). Case 2 (**C**): axial FLAIR-weighted MRI showing multiple subcortical white matter lesions. Case 3 (**D**): axial FLAIR-weighted MRI showing no alterations. Case 4 (**E**): sagittal T1-weighted spin echo MRI showing cerebellar atrophy. Case 6 (**F**): axial FLAIR-weighted MRI showing subcortical white matter lesions. Case 7 (**G**): sagittal FLAIR-weighted MRI showing mild cerebellar atrophy. Case 8 (**H**–**I**): axial (**H**) and sagittal (**I**) FLAIR-weighted MRI showing atrophy of the middle cerebellar peduncle and pons

Four females and two males were found. The youngest patient (case 3) was 44 years of age, and the oldest (case 5) was 79 years. Disease duration spanned from 4 to 22 years, and clinical presentation and progression was greatly different among patients (Fig. 2) (Table 3). Three of the six patients met the criteria [34] for probable FXTAS and one for possible FXTAS (Table 4). Five out of six patients had at least one major clinical criteria: four cerebellar gait ataxia and one cerebellar ataxia and intention tremor. Minor clinical criteria were represented as follows: parkinsonism was present in one patient, cognitive decline in two cases, and neuropathy in one case. None of the patients presented the major radiological criteria, but four out of six had one or more minor radiological criteria (Table 4) (Fig. 3). Family history for neurological disorders was positive in two cases, in which one of the parents was affected by dementia.

**Table 3:** Phenotypic characteristics of the FMR1- intermediate allele carriers

Case	Age at onset	Sex	Disease duration	Family history	Number of triplets	First symptoms	Cerebellar symptoms	Neurological not cerebellar symptoms	Other diseases
1	60 y	F	6 y	No	FMR1: 45 SCA2: 28	Cognitive decline	Ataxic gait	Behavioral symptoms	Positivity for ANA autoantibody
2	67 y	F	4 y	Yes (cognitive decline father; anxiety-depressive disorder sister)	FMR1: 48	Ataxic gait	Dysarthria Dysmetria Dysdiadochokinesia		Positivity for anticardiolipin autoantibody
3	40 y	F	6 y	No	FMR1: 45	Nystagmus			
4	44 y	M	20 y	No	FMR1: 46	Ataxic gait	Dysmetria Dysarthria Dysphagia Oculomotor alterations Dysdiadochokinesia	Sensory neuropathy	
5	78 y	M	4 y	No	FMR1: 52	Ataxic gait	Dysmetria Dysarthria Dysphagia Intentional tremor Oculomotor alterations	Cognitive decline	Atrial fibrillation
6	52 y	F	7 y	Yes (cognitive decline, probable Alzheimer’s disease mother)	FMR1: 54	Nystagmus	Ataxic gait Dysmetria	Parkinsonism, Pyramid signs, autonomic dysfunction	Homozygosis mutation for MTHFR, previous deep vein thrombosis, patent foramen ovale, headache, glaucoma

**Table 4:** Evaluation of the clinical and radiological diagnostic criteria of FXTAS in the six FMR1-intermediate allele carriers

	Clinical criteria		Radiological criteria		Diagnostic categories^a^		
	Major -Cerebellar gait ataxia -Intention tremor	Minor -Parkinsonism -Neuropathy -Memory and executive function deficits	Major -White matter lesions in the middle cerebellar peduncles (MPC sign) or brainstem	Minor -White matter lesions in the splenium of the corpus callosum -Cerebral white matter lesions -Moderate-severe brain atrophy	Definite	Probable	Possible
Case 1	**X**	**X**		**X X**		**X**	
Case 2	**X**			**X**			
Case 3							
Case 4	**X**	**X**					**X**
Case 5	**XX**	**X**		**X**		**X**	
Case 6	**X**	**X**		**X**		**X**	

^a^Diagnostic categories [34]. Definite: 1 clinical major + 1 radiological major OR neuropathological major; Probable: 2 clinical major OR 1 clinical minor + 1 radiological major; Possible: 1 clinical major + 1 clinical minor

#### SCA2 (27–33 CAG Repeats) [30, 31]

Case 1 was found to be also a carrier of an IA in the ATXN2 gene. The patient presented cognitive and behavioral disorder associated with mild cerebellar gait. MRI presented white matter alterations and diffuse brain atrophy (Fig. 3). FTD-related genes were negative for mutations. Family history was negative for neurological disorders (Tables 3 and 4).

#### SCA8 (50–80 CTG Repeats) [27]

These were two females. Case 7 was 60 years old at disease onset and presented a cerebellar syndrome characterized by gait ataxia, intention tremor, dysmetria, dysarthria, nystagmus, urinary urgency, and panic disorder. Case 8 was diagnosed as having probable MSA-C due to the presence of cerebellar syndrome, parkinsonism, and autonomic failure associated with atrophy of the middle cerebellar peduncle and pons at brain MRI. Presynaptic dopaminergic denervation was detected with brain ([123I]FP-CIT SPECT) (Fig. 3).

### Patients with Full-Range Expansion

#### FXTAS (> 55 CGG Repeats) [29]

The patient with full-range expansion of the FMR1 gene (92 triplets) was a 64-year-old male. Symptoms started with very mild intention tremor of the hands at the age of 52 years. After 4 years, he presented orthostatic hypotension and mild sensory neuropathy. Gait ataxia started 9 years after intention tremor at the age of 61 years. The disease progressed slowly and the SARA score was 4 after 12 years from symptoms onset. Cognition was normal. Brain MRI showed white matter lesions in the middle cerebellar peduncles. The patient fits criteria for definite diagnosis of FXTAS [34].

#### SCA2 (> 33 CAG Repeats) [30, 31]

These were two males with typical clinical phenotype. The disease started with ataxic gait at the age of 51 years in one case (39 triplets) and at the age of 45 years in the other (40 triplets). In both cases, the disease progressed with balance impairment, dysarthria, dysmetria, and oculomotor alteration such as jerky pursuit and increased latency of saccadic horizontal movements. Seven years after the onset of symptoms, the SARA score was 7 in one case and 12 in the other. Cognitive performance was preserved in both cases.

## Discussion

We explored the etiology of CA in a cohort of 66 adult, unrelated patients recruited over a period of 5 years at the Neurological Department of the University of Florence, Italy. The most frequent diagnosis was SAOA, followed by MSA-C. Acquired ataxia covered 9% of cases, such as genetic full-range expansion cases. We found eight out of the 66 patients (12%) carrying an IA. The IAs were six in the FMR1 gene, two in the SCA8 gene, and one in the ATXN2 gene. We did not find subjects carrying IA in the genes associated with the other SCAs. Few previous studies attempted to systematically investigate the causes of CA in cohorts of patients clinically presenting sporadic adult-onset cerebellar syndromes [35–38]. The comparison between studies is difficult because of differences in diagnostic category classification; however, neurodegenerative acquired forms, MSA-C and SAOA, were the most frequent in all cohorts, except in one [37]. MSA-C had a frequency ranging from 36 [35] to 69% [36]. In our cohort, MSA-C was 18% of cases, but we included only patients with probable forms. The percentage of acquired ataxias confirmed what was reported in a comparable sample size French cohort (8.75%) [35], and it is higher than the 5% identified in two wider cohort-studies [36–38]. Only Hadjivassiliou and colleagues reported 38% of acquired ataxia in UK patients affected by CA. The cases were mostly represented by gluten ataxia. The high percentage of gluten ataxia described by these authors is unique worldwide and depends in part on the different definition of the disorder given by the authors, with the inclusion of cases without bowel coeliac manifestation and with lower antibody titer [37]. Full-range expansion was identified in 9% of cases, similar to the frequency of 7 to 12% reported in the other cohort studies [35–38]. We found, in addition, a high percentage of cases associated with IAs. Evaluation of the frequency of IAs among patients with CA is reported only for the FMR1 gene and only in three studies [18, 20, 36]. The percentage identified in those studies did not differ from the one reported in the general reference population [18, 39]: 1.5%. Data on the frequency of IAs in SCAs genes among patients with CA are, instead, not available.

### Intermediate Alleles in FMR1 Gene

FXTAS was first described in 2001 [40] with a clinical picture classically represented by intention tremor, cerebellar gait ataxia and parkinsonism, associated with hyperintensity of middle cerebellar peduncles at brain MRI [41] and the premutation of the FMR1 gene (55–200 triplets). Typical age at onset is around 60 years; males are more frequently affected than women with a more severe clinical picture [34]. Clinical picture is heterogeneous with a disease duration ranging from 5 to 25 years. Next to ataxia, tremor, and parkinsonism, the following symptoms are frequently detected among FXTAS patients: cognitive decline, neuropathy, autonomic dysfunction, eye gaze abnormalities, and psychiatric and sleep problems. Migraine headache, autoimmune diseases, fibromyalgia, and primary ovarian failure are common in females with FXTAS [34]. In 2012, Hall and colleagues [12] reported for the first time a case series of three patients (one man and two women) with intention tremor and other signs typical of FXTAS that were carriers of gray zone FMR1 allele. Two other cases were reported by Liu [17]. At biological level, IAs patients presented the same molecular changes as premutation carriers: an increased level of the FMR1 mRNA that is supposed to determine a neurotoxic effect [42]. Therefore, the presence of IA was admitted as genetic criteria in the revised diagnostic criteria of FXTAS [43]. In our cohort, four out of the six patients met the criteria for FXTAS, three probable and one possible. Two females (case 2 and case 3) did not meet the criteria. Their disease duration was 4 and 6 years, respectively. Case 2 presented a complete cerebellar syndrome (gait ataxia, dysmetria, dysarthria), while case 3, despite a subjective sensation of disequilibrium, presented only oculomotor abnormalities (vertical and horizontal nystagmus and diplopia) (Fig. 2). It is interesting to note that case 3 had the same initial clinical presentation as case 6. In fact, case 6 started with a 4-year history of downbeat nystagmus and diplopia, followed by the other cerebellar symptoms, parkinsonism, and autonomic failure. Probably, the clinical pictures of case 2 and case 3 will become completely manifest in the coming years. Disease duration in our cohort was comparable to what is reported in the literature in the patient with the premutation. The most frequent symptom was gait ataxia, followed by eye gaze abnormalities, while parkinsonian symptoms were present only in one case. That differs from most of the data from the literature in which the FMR1 gray zone allele has been reported as risk factors for PD and parkinsonism in both females and males [13–16, 18].

In our cohort of patients, the frequency of the IA in the FMR1 gene was higher than that found in our control cohort (1.42%) and then that previously reported in the general population ranged between 0.3 and 2.6% [44, 45]. However, an estimation of the prevalence of FMR1 gray zone allele among the Italian population has never been reported. Our patients met the criteria for FXTAS but not with typical presentation, and the lack of epigenetic analysis on FMR1 and the lack of data on the FMR1 mRNA level [42, 46] do not allow us to be conclusive on the role of the IA in the pathogenesis of their symptoms.

### Intermediate Alleles in ATXN2 Gene

One patient (case 1) had IAs for both FMR1 and ATXN2 genes. SCA2 is one of the most frequent forms of SCA in Italy, and around 2.7% of healthy Italian subjects are carriers of the IA [4]. Typically, SCA2 is characterized by the presence of ataxic gait, impaired balance and speech, dysmetria, and slow eye movements, as described for our two patients with full-range expansion, but the clinical spectrum can also include parkinsonism [47] and cognitive decline with impairment of executive functions, memory, and visuospatial skills [48]. Recent data support the hypothesis that ATXN2-IA increased the risk of amyotrophic lateral sclerosis (ALS) and of frontotemporal dementia [7, 9]. Other phenotypes described in association with the ATXN2-IA were one case of ALS with cerebellar syndrome [8] and one patient with MSA [6]. Our case presented cognitive decline, associated with balance impairment and behavioral symptoms but neither parkinsonism nor motor neurons involvement. In this patient, given the peculiar genetics, in the absence of biomolecular analysis or neuropathological data, it is not possible to determine whether and how the symptomatology can be traced back to alteration on the FMR1 gene or on ATXN2 gene. In any case, the patient met the criteria for probable FXTAS. Despite cases of SCA2 full expansion were as frequent as expected in our cohort [4], we did not identify any IA in the control cohort. The frequency of the IA was comparable between the study population and the control cohort. This supports the hypothesis that the IA of ATXN2 is not associated with ataxic phenotype, even in our cohort..

### Intermediate Alleles in SCA8 Gene

The clinical presentation of SCA8 is typically characterized by late-onset slowly progressive cerebellar ataxia occurring alone or with other symptoms, such as upper motor neuron dysfunction, peripheral sensory disturbance, or psychiatric symptoms [49, 50]. Here, we reported one patient carrying an IA with 78 repeats that presented a typical ataxic phenotype. Previous studies identified IAs both in families and in sporadic cases with cerebellar ataxia [51]. Our second patient with IA fit the criteria for probable MSA-C [25]. Recently, non-ataxic phenotypes have been described in patients bearing both the expanded allele and the IA at SCA8 locus, such as ALS, Parkinson disease [52], and atypical parkinsonism [10]. In one of the two previously reported probable MSA cases with SCA8 expansion [11, 19], the neuropathological data confirmed the diagnosis of MSA [19]. Those data and the similar frequencies of expanded alleles found in healthy controls and in patients [53] raised questions about the pathogenetic role of SCA8 expansion. Transgenic murine models [54] developed an ataxic phenotype; however, due to the reduced penetrance of the mutation, in the absence of neuropathological data, caution is needed in the evaluation of patients bearing SCA8-IA or expanded allele. In two previous studies on Italian ataxic patients, the frequency of the SCA8 full-range expansion was 0.88% [55] and 02.9% of the cases [56]. In this cohort, this IA represents (2/66) 3% of cases. There is no previous estimate on the frequency of SCA8-IA in the general population, neither in Italy nor worldwide. In our control cohort, we did not identify any expansion at the SCA8 genetic locus.

## Conclusion

To our knowledge, this is the first study to systematically search for genetic expansions in the intermediate range in genes responsible for the major SCAs and for FXTAS among patients with CA. The present cohort resulted comparable to the previous ones in terms of frequency of the different causes of the cerebellar syndromes; nevertheless, as an original finding, there was a high percentage of patients bearing an IA. IA carriers presented phenotypic features similar, in some respects, to those described in subjects with the corresponding pathological expansion. IAs pose a challenge in genetic counseling and our data promote further investigations aimed at assessing the frequency of IAs both in the general population and in subjects with ataxia.

### Supplementary Information

Below is the link to the electronic supplementary material.

Supplementary file1 (DOCX 19 KB)

## Data Availability

Data from this study are available from the corresponding author upon reasonable request.
