# Supplementary material for: The Frequency of Intermediate Alleles in Patients with Cerebellar Phenotypes
Source: Cerebellum. 2023 Oct 31;23(3):1135–45. doi: 10.1007/s12311-023-01620-7 (PMC11102406; doi:10.1007/s12311-023-01620-7)
Supplement: Supplementary file 1 — Supplementary file1 (DOCX 19 KB) [file 12311_2023_1620_MOESM1_ESM.docx]

**Supplementary Materials**

**Case 1**

A 64-year old woman presented slight ataxic gait and balance impairment (SARA scale=4), preceded by a 4-years history of memory disorder, difficulties in recognizing familiar faces and language impairmentt with difficulty in finding words. At the time of evaluation MMSE was 13/30. In addition to cognitive symptoms, she presented depression with emotional lability. She performed brain MRI that showed ,in the T2 and FLAIR sequences, hyperintensity of the subcortical white matter, of the semioval centers and of the superior cerebellar peduncles with extension to the posterior portions of the midbrain Diffuse brain atrophy was also detected (**Figure 3**). The analysis of cerebrospinal fluid was normal including the dosage of the neurodegenerative biomarkers. After six months there was a slight progression of both cognitive and motor symptoms, and she scored 12/30 on MMSE and 5 on SARA scale. Family history was negative for neurological disorders.

Genetic tests identified intermediate alleles in the SCA2-gene (28 triplets) and in theFMR1 gene (45 triplets). Due to the presence of cognitive and psychiatric symptoms she also underwent genetic test for genes associated with Frontotemporal dementia (C9orf-72, MATP, Progranulin), that were negative for mutations.

**Case 2***.*

A 68-yearold woman referred 2 years of slight balance impairment. At the neurological examination she presented ataxic gait, mild dysmetria of the four limbs with prevalence at the left arm, suggestion of dysarthria, absence of tremor at rest, postural or intentional, nor bradykinesia or muscle tones alteration. Her score on the SARA scale was 5. MRI showed white matter lesions of the semioval centers, but not brain atrophy (**Figure 3**). The vestibular examination detected a mild impairment of the floccular area of the cerebellum. Neurophysiological investigations were normal, as was dysautonomic screening. Blood tests showed positive results for inflammatory indices (CRP) and anti-cardiolipine antibodies in the absence of reumathological symptoms. Symptoms worsened rapidly and after 18 months gait was staggering with difficulties in half turn, standing was no longer possible with feet togheter, dysarthria was evident, as well as dysmetria and dysdiadochokinesis, and her score on the SARA scale was 12. Tremor, rigidity and bradykinesia were still not detected, as well as there was no cognitive impairment. Family history was positive for neuropsychiatric diseases: her father had cognitive decline at the age of 78 years, and her 5-years older sister had anxiety-depressive disorder. Genetic tests identified a FMR1 intermediate allele (48 triplets).

**Case 3.**

A 44-year old woman complained balance impairment, inconstant diplopia and oscillopsia, the latter having been present for some years. On neurological examination, the only notable finding was the presence of bilateral horizontal and vertical inexhaustible nystagmus. The vestibular examination confirmed the dysphunction of the floccular and nodular areas of the cerebellum and of the midbrain. Brain MRI was normal. After two years the patient was clinically stable (SARA score =0). Family history was negative for neurological disorders. Genetic tests revealed the presence of 45 triplets at the level of the FMR1 gene.

**Case 4**.

A 64-year old man presented a twenty years history of gait ataxia with slow progression associated with sensitive neuropathy. At the neurological examination he presented ataxic gait with wide base possible only with support, dysmetria of the four limbs with prevalence at right arm and leg and dysarthria. He had bilateral exhaustible horizontal nystagmus. Muscle tone was normal in the upper limbs, slightly increased in the lower limbs. Intentional, postural or at rest tremor was not detected. He reported occasional dysphagia . He scored 13 on SARA scale. Brain MRI showed cerebellar atrophy with volumetric reduction of the brainstem and the presence of multiple periventricular gliotic areas. The vestibular examination showed clear signs of vestibular dysfunction at brainstem level with bilateral hyporeflexia and preponderance of the left hemi-system. Cognitive performance was normal. Neurological examination was stable after six months. Family history was negative for neurological disorders. Genetic tests identified aFMR1 intermediate allele (46 triplets).

**Case 5.**

A79-year old man referred two years of progressive gait ataxia, associated, after one year, with dysarthria and dysphagia. On the neurological examination he presented gait ataxia, with postural instability. Stance was possible only with wide base. Frenage was bilaterally detected at upper limbs, as well as bilateral intentional tremor. He had mild dysmetria of the lower limbs, while positional or at rest tremor were not present. The patient also had oculomotor alteration, with jerky pursuit and increased latency of saccadic horizontal movements. The speech was scanning but understandable. The SARA score was 13. Cognitive test detected mild memory and executive function impairment, with a score at MMSE of 23/30. Brain MRI showed severe cerebellar and cerebral atrophy. Neurophysiological investigations were normal. Genetic test identified a FMR1 intermediate allele (52 triplets).

**Case 6.**

A 58-year old woman presented at the age of 52 years a rapid onset of diplopia and oscillopsia. Symptoms were stable for four years. Then, the patient complained progressive balance impairment. The neurological examination showed staggering wide base gait, down beating nystagmus and diplopia in all directions. Muscle tone and tendon reflex were normal on the four limbs, and she performed the cerebellar coordination tests correctly. Her SARA score was 7. Symptoms worsened and after six months her neurological examination showed marked staggering gait possible only with monolateral support, stance without support but with very wide base, bradykinesia of the left hemisoma, dysmethria of the left limbs, and slight increased of muscle’s tone on the left lower limb. Speech was normal and tremor was not present. Pyramidal signs were also detected: increased tendon reflex at the four limbs and left Babinski. Her score on SARA scale was 12. The patient also reported the recent occurrence of urinary incontinence. MRI showed subcortical white matter lesions, but not cerebral atrophy. SEP, MEP and EMG did not detect any alteration. Family history was positive for neurological disorder: the mother had a cognitive decline classified as probable Alzheimer disease. Genetic test detected a FMR1 intermediate allele (54 triplets).

**Case 7***.*

A 60-year old woman presented postural instability and “drunken sailor” gait, walking was possible only with bilateral support; she also had bilateral intention tremor at the upper limbs, dysmetria at coordination tests and dysdiadochokinesis. Spasticity and hypertonia were detected in the lower limbs, while muscle tone was normal in upper ones. Muscular strength was preserved. The patient also presented a scanning dysarthria, a bilateral horizontal inexhaustible nystagmus, jerky pursuit. The specialist audologist objected: down beating nystagmus; gaze-evoked nystagmus; rebound nystagmus; perverted-Head Impulse Test; impaired smooth pursuit; impaired optokinetic reflex; decreased ability to suppress the vestibulo-oculomotor reflex during fixation of an object rotating with the patient’s head and to suppress caloric nystagmus by fixating a stationary target. SEP, MEP and EMG did not detect any alteration. Urinary urgency, anxiety and panic disorder were also present. The patient had not parkinsonian features. Her score on the SARA scale was 17. The symptomatology had started two years earlier with difficulties in balance and walking. Brain MRI was performed, showing a mild cerebellar atrophy, DAT-scan was negative for any dopamine deficiency. After 24 months the patient was clinically stable, SARA score was 18. Family history was negative for neurological disorder.

The genetic analysis detected an intermediate allele, with 78 repetitions, at the level of the gene responsible for SCA8.

**Case 8.**

A 47-year old woman complained since the age of 45 years balance instability and vertigo. Balance impairment got worse rapidly and was associated with internal tremor and tension. After a year urinary incontinence was also present. On the neurological examination she showed wide-based walking with small and short steps, resting tremor, bradykinesia and rigidity of the left hand. Balance was impaired with increased risk to fall. Pyramidal signs and bilateral horizontal inexhaustible nystagmus were also detected. Brain MRI showed atrophy of the middle cerebellar peduncle and pons, and DAT-SCAN detected a presynaptic dopaminergic denervation (distribution volume: caudate right 1.56 (2.58 +-0,41) caudate left 1.97 (2,57+- 0.37), putamen right 1.03 (2.14+-0.41), putamen left 1.25 (2.23+-0.45) (Fig 3). Therapy with L-DOPA was started, without any clinical benefit. Based on clinical and instrumental results a diagnosis of Probable MSA-C was made. Anyhow she was also screened for genes associated with ataxic disorders and was found to be a carrier of an intermediate allele in the gene responsible for SCA8 (61 tripltes). Family history was positive for neurological disorders: maternal grandmother was affected by Parkinson disease.
